# Quo Vadis Gerotranscendence? A Systematic Literature Network Analysis of Existing Themes and Emerging Trends in Gerotranscendence Theory with a Focus on International Applications

**DOI:** 10.1007/s10943-025-02349-9

**Published:** 2025-06-02

**Authors:** Deniz Pamuk, Sanam Asadi Faezi, Mehmet Efe

**Affiliations:** 1 Department of Gerontology, Muş Alparslan University, Muş, Turkey; 2 Department of Gerontology, Akdeniz University, Antalya, Turkey

**Keywords:** Gerotranscendence, Tornstam, Gerontological theory, Gerontological nursing

## Abstract

Lars Tornstam’s Gerotranscendence Theory suggests that the aging process involves a shift in perspective from materialistic concerns to a more cosmic outlook, characterized by transcendence in cosmic, personal, and social dimensions. This study uses a Systematic Literature Network Analysis to explore the central themes and emerging trends in gerotranscendence-related research. A total of 139 articles published between 1992 and 2024 were retrieved from the ISI Web of Science database. The findings revealed core thematic areas including aging care, religiosity, and reminiscence, along with emerging areas such as marketing, environmental design, and gerontology. Although the theory originated in Sweden, its application has become increasingly global, with significant research in countries such as India, China, and Turkey. Despite its relatively recent development, gerotranscendence demonstrates considerable potential as a conceptual framework for interventions that promote positive aging.

## Introduction

Swedish gerontologist Lars Tornstam introduced the concept of Gerotranscendence, which suggests that, with aging, individuals’ worldviews gradually shift from a materialistic and pragmatic orientation to a more cosmic and transcendent perspective (Tornstam, 1997). Gerotranscendence Theory represents a relatively recent development within psychological theories of aging (Schroots, 1996). It emphasizes three key dimensions of developmental transformation across the lifespan: cosmic transcendence, self-transcendence, and social transcendence (Tornstam, 2005, 2011).

The first dimension, cosmic transcendence, involves altered perception of time, an increased sense of connection with past generations, diminished fear of death, and acceptance of the finite nature of life. Self-transcendence, the second dimension, refers to a growing awareness of previously unrecognized aspects of the self, a reduction in egocentrism, and a shift toward transcending concerns related to the body, ego, and personal identity. Tornstam (2011) noted that whereas Erikson’s (1982) concept of ego integrity includes accepting both the positive and negative aspects of one’s life, ego transcendence, central to gerotranscendence, focuses primarily on the positive reinterpretation of life experiences.

The third dimension, social transcendence, encompasses shifts in the meaning and importance of interpersonal relationships, changes in social roles, the development of a sense of emancipated innocence, the adoption of modern asceticism, and the attainment of everyday wisdom with a transcendent quality.

Although an increasing number of studies have examined various dimensions of gerotranscendence, there remains a notable gap in the literature concerning its practical applications and the specific domains in which it is most frequently investigated. Gaining a comprehensive understanding of the current research landscape is essential for identifying future research priorities. Accordingly, this article aims to provide a quantitative and visual synthesis of existing studies on gerotranscendence through the use of Systematic Literature Network Analysis (SLNA). Specifically, it seeks to address the following research question: What is the current scope of research on Gerotranscendence Theory?

## Methods

Systematic Literature Network Analysis (SLNR) is an emerging methodology that integrates systematic literature reviews techniques with citation network analysis, as originally proposed by Colicchia and Strozzi (2012).

**Fig. 1:**
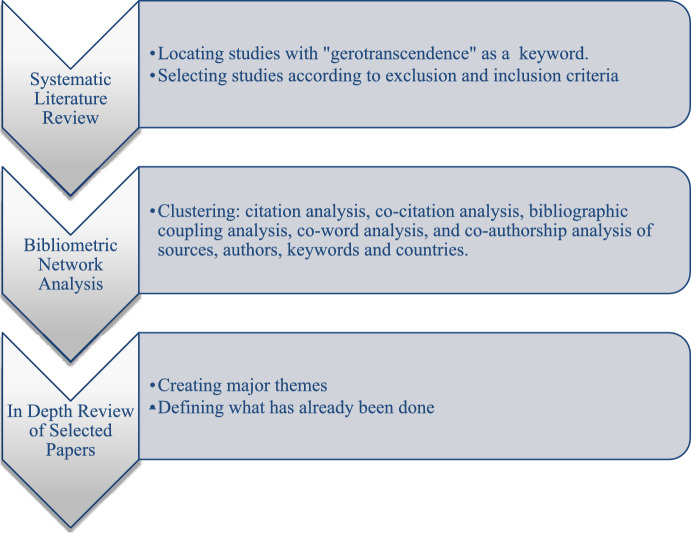
Flow diagram representing the SLNA process

Its primary objective is to examine the processes of knowledge creation, transfer, and evolution from a dynamic, systems-oriented perspective. While bibliometric network analysis reveals the impact and dissemination patterns of theoretical frameworks, a systematic literature review identifies key research themes and illustrates the extent to which a theory is represented across various subfields of gerontology. Figure 1 shows the overall workflow of the SLNR approach utilized in this study.

For this study, data were retrieved from the ISI Web of Science database using the search term “gerotranscendence.” Given that gerotranscendence is a relatively recent theoretical framework, no restrictions were placed on the time span of the search. The search was conducted on October 10, 2024, within a single day to minimize potential bias arising from daily updates to the database.

Articles were included in the analysis if the term “gerotranscendence” appeared in the title, abstract, or keywords. An initial total of 173 studies was identified. Study selection and data extraction were conducted independently by the authors, who then compared and reconciled their final selections. Following the application of predefined inclusion and exclusion criteria, the final sample was narrowed to 139 articles.

### Inclusion–Exclusion Criteria

**Fig. 2:**
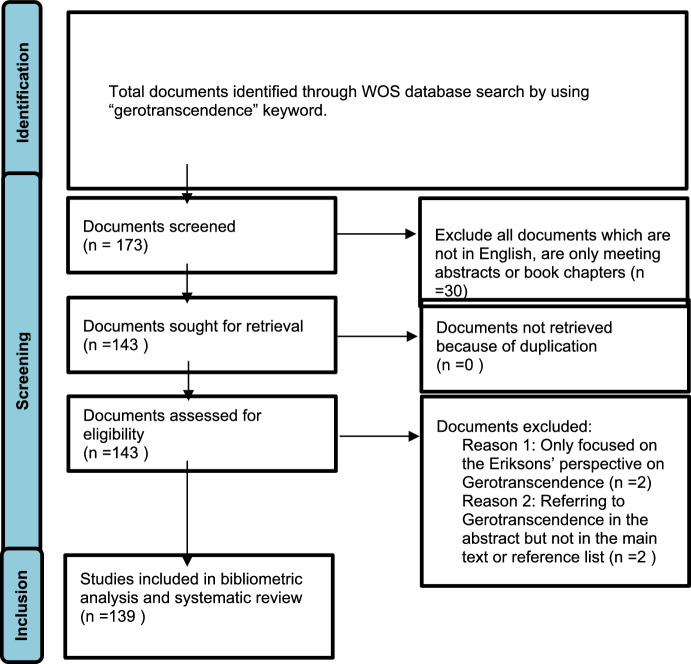
PRISMA flowchart for bibliometric analysis and systematic review of gerotranscendence

This study included publications specifically focused on Lars Tornstam’s Gerotranscendence Theory. As the analysis was centered exclusively on Tornstam’s conceptualization, studies referencing only Erikson and Erikson’s (1997) version of gerotranscendence were excluded. Only peer-reviewed journal articles published in English were included in the final dataset. The PRISMA flowchart presented in Fig. 2 outlines the detailed steps of the article selection process.

### Data Analysis

Data analysis and visualization were conducted using the Biblioshiny extension of the Bibliometrix R package (Aria & Cuccurullo, 2017) and VOSviewer (Van Eck & Waltman, 2014). After the bibliometric analysis and systematic review of the selected publications, study themes were identified using a phenomenological approach. The authors conducted a detailed examination of the titles and content of the articles retrieved from the Web of Science (WOS) database, deriving thematic categories based on recurring patterns and conceptual similarities observed across the literature.

## Results

### General Characteristics of the Publications

After applying the inclusion and exclusion criteria, 139 publications were selected for analysis. The final dataset spans the period from 1992 to 2024. The earliest publication, authored by Tornstam (1992), introduces the foundational principles of gerotranscendence theory. The most recent study, by Tip et al. (2024), explores how nursing home residents perceive and recognize signs of gerotranscendence.

**Fig. 3:**
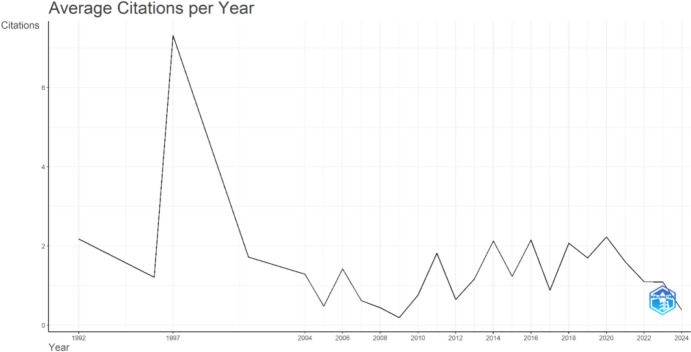
Average citations of publications on gerotranscendence

The annual distribution of scientific publications on gerotranscendence is presented in Fig. 3. The trend demonstrates a fluctuating pattern over the years; however, a notable upward trajectory has been observed since 2012. The peak in publication frequency occurred in 2023, with 17 articles, followed by 2019 and 2021, each with 10 publications.

**Fig. 4:**
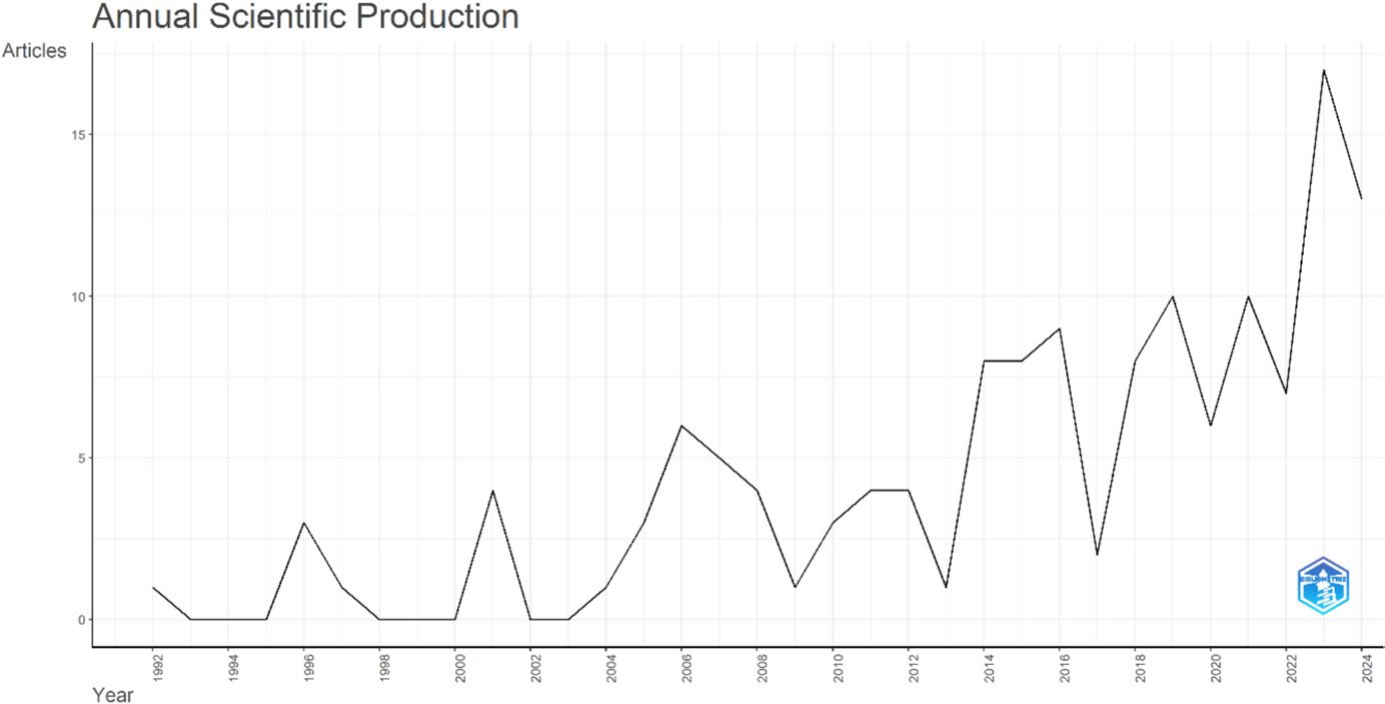
Annual scientific production of publications on gerotranscendence

Figure 4 presents the annual total number of citations received by all publications analyzed in the study on gerotranscendence.

The highest number of citations related to gerotranscendence was recorded in 1997, followed by a peak in 2020. This trend suggests a marked increase in scholarly interest in the theory during these periods.

## Research Area

Table 1 presents the journals that have published the highest number of articles on gerotranscendence. When categorized by research discipline, the majority of these publications—78 in total—fall within the field of Geriatrics and Gerontology.

**Table 1:** Most productive research areas in gerotranscendence

Rank	Field	Number of Publications
1	Geriatrics/Gerontology	78
2	Nursing	27
3	Psychology	23
4	Psychiatry	11
5	Religion	7
6	Education—Educational Research	8
7	Public Environmental / Occupational Health	5
8	General Internal Medicine	2
9	Dentistry—Oral Surgery Medicine	2
10	Linguistics	2

This is followed by publications in the fields of nursing, psychology, psychiatry, religion, education and educational research, public, environmental, and occupational health, general internal medicine, dentistry, and linguistics.

In the field of dentistry (oral surgery and medicine), studies have demonstrated a negative association between gerotranscendence scores and oral health-related quality of life among older adults in Japan (Mihara et al., 2018) and India (Das et al., 2023).

Meanwhile, two publications in the field of linguistics have investigated the relationship between humor and levels of gerotranscendence in samples of older Polish adults (Brudek et al., 2021b; Brudek & Stauden, 2022). These studies provide empirical support for specific subdimensions of gerotranscendence, particularly body transcendence and emancipated innocence, and illustrate how the theory extends beyond the health sciences into the realm of the linguistic and cultural domains.

## Keywords

**Fig. 5:**
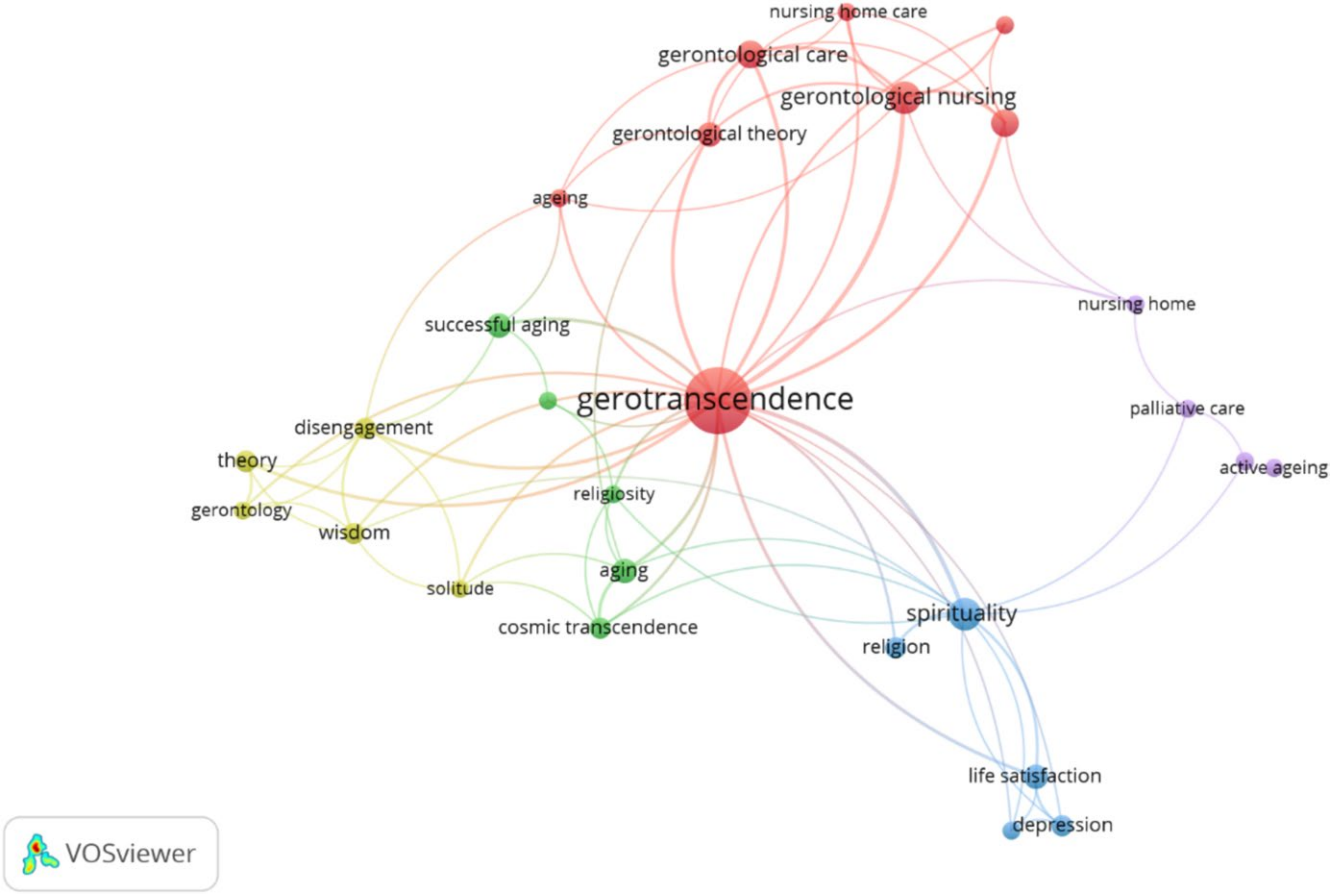
Co-occurrence networks of the keywords

Figure 5 illustrates the co-occurrence network of keywords related to gerotranscendence research. In this network, strongly connected keywords appear closer together, while weakly connected keywords are positioned farther apart. The resulting colored clusters represent distinct subfields within gerotranscendence, as conceptualized through keyword associations (Boyack & Klavans, 2010).

Gerotranscendence occupies the central position on the keyword co-occurrence map, signifying its foundational role and strong connections to all other identified terms. The size of each circle corresponds to the frequency with which each keyword appears in the analyzed studies.

The red cluster contains highly prevalent keywords, including “nursing home care,” “gerontological nursing,” “gerontological care,” “gerontological theory,” and “aging,” indicating a dominant focus on care-related and theoretical aspects within the field. In contrast, the yellow cluster encompasses keywords such as “disengagement,” “solitude,” and “wisdom,” reflecting a thematic emphasis on the introspective and philosophical dimensions of aging.

The blue cluster represents themes related to spirituality, encompassing keywords such as religion, life satisfaction, and depression. This grouping reflects the psychological and existential dimensions of gerotranscendence as they relate to spiritual well-being.

The green cluster includes keywords such as religiosity, cosmic transcendence, and successful aging, highlighting the theoretical and aspirational aspects of aging as conceptualized in gerotranscendence theory.

### The Most Cited Publications and Overall Themes

According to Table 2, three of the ten most cited publications—specifically the first, fourth, and ninth—are authored by Lars Tornstam (1992, 1996, 1997). In his 1992 article, Tornstam critiques the dominant “misery perspective” of old age and introduces the concept of gerotranscendence. In 1996, he proposes gerotranscendence as a valuable framework for elder care. By 1997, Tornstam further elaborates on the theory, positioning it as an alternative approach to understanding the psychosocial dimensions of aging.

**Table 2:** Ten most cited publications on gerotranscendence

Rank	Web of science citations	Author	Title	Year
1	205	Tornstam	Gerotranscendence: the contemplative dimension of aging	1997
2	88	Adams	Depressive symptoms, depletion, or developmental change? Withdrawal, apathy, and lack of vigor in the geriatric depression scale	2001
3	84	Dalby	Is there a process of spiritual change or development associated with ageing? a critical review of research	2006
4	72	Tornstam	The quo-vadis of gerontology—on the scientific paradigm of gerontology	1992
5	75	van Dyk	The appraisal of difference: Critical gerontology and the active-ageing-paradigm	2014
6	56	Balducci	Geriatric oncology, spirituality, and palliative care	2019
7	54	Nyman & Szymczynska	Meaningful activities for improving the wellbeing of people with dementia: beyond mere pleasure to meeting fundamental psychological needs	2003
8	53	Carver & Buchanan	Successful aging: considering non-biomedical constructs	2016
9	47	Tornstam	Caring for the elderly—introducing the theory of gerotranscendence as a supplementary frame of reference for caring for the elderly	1996
10	47	Braam et.al	Cosmic transcendence and framework of meaning in life: Patterns among older adults in The Netherlands	2006

Dalby (2006) reviewed 13 articles related to spiritual change and gerotranscendence, concluding that although Tornstam did not explicitly address religion in his theory, research indicates that the paradigm shift also occurs at the religious level. Adams (2001) applied the social disengagement and decrease activity aspect of Gerotranscendence Theory, emphasizing the importance of considering this factor when assessing depression in older adults. Similarly, Carver and Buchanan (2016) proposed gerotranscendence as an alternative framework for evaluating successful aging. Additionally, van Dyk (2014) presented gerotranscendence as an alternative to active aging from a critical gerontology perspective. Braam et al., (2006 explored the relationship between cosmic transcendence and the search for meaning in the life patterns of older adults in the Netherlands. Finally, Balducci et al. (2019) reviewed the connection between cancer and gerotranscendence.

### Authors

#### Co-Authorship

Co-authorship serves as an indicator of both national and international collaboration among authors. This metric is used to define collaborations in publications authored by at least two individuals (Okubo, 1997). Increased collaboration between authors signifies stronger relationships and can be seen as a potential for future partnerships (Boyack & Klavans, 2010). Braam and Deeg have co-authored two publications: one exploring cosmic transcendence (Braam et al., 2006) and the other investigating the relationship between life crises and gerotranscendence (Read et al., 2014). Similarly, Calatayud and Tomas have co-authored two publications on successful aging (Gutiérrez et al., 2018a, 2018b; Gutiérrez, Calatayud & Tomas, 2018).

#### Co-Citation

Co-citation analysis indicates that two publications are connected when they appear in the reference list of a third publication (Donthu et al., 2021). Tornstam, Wadenstein, and Erik Erikson were co-cited with at least 20 of the 139 articles analyzed. This finding suggests that these scholars may be foundational to the body of research on gerotranscendence.

### Countries

**Fig. 6:**
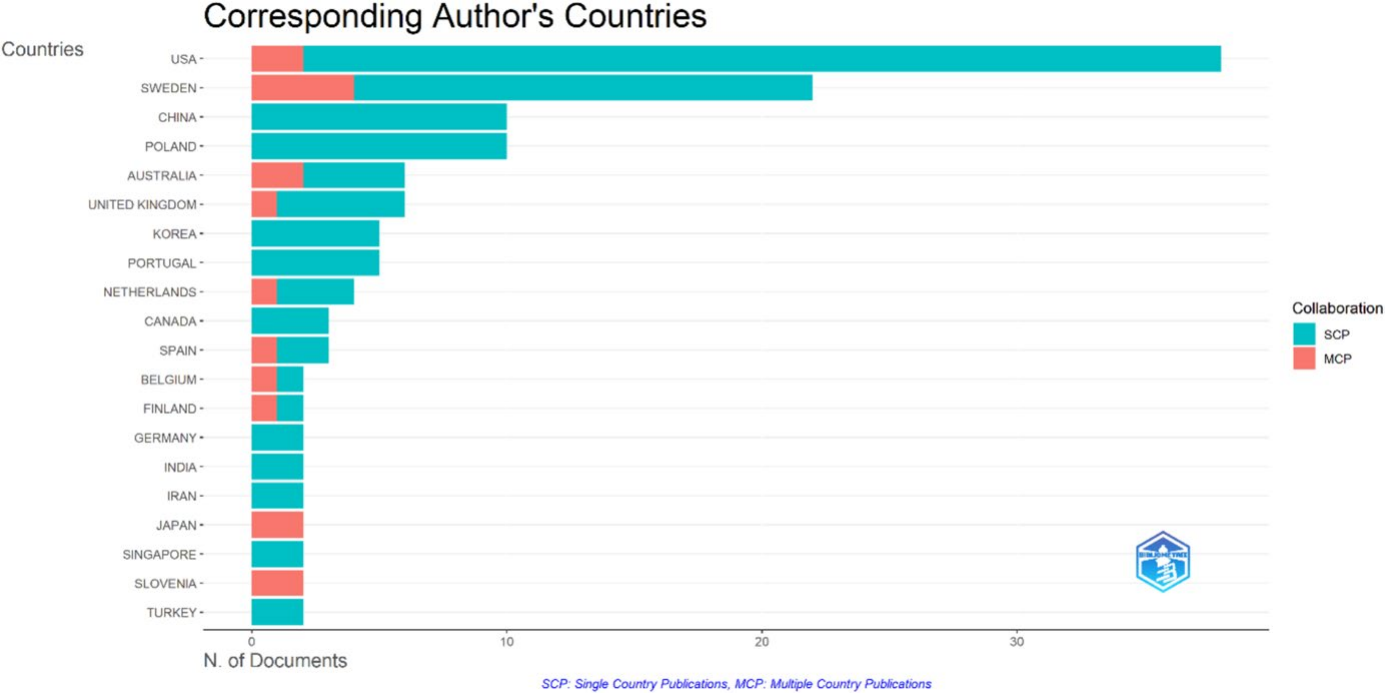
Corresponding authors’ countries with distributions of intra-country (SCP) and inter-country (MCP) collaborations

Figure 6 illustrates the distribution of countries for the corresponding authors.

Figure 6 indicates that the USA, Sweden, China, and Poland are the leading countries in gerotranscendence research. However, the graph also highlights that the theory’s scope extends to a wide range of countries, including Korea, Portugal, India, Iran, and Turkey.

### Sources

Table 3 lists the ten most productive journals.

**Table 3:** Most productive journals in Gerotranscendence

Rank	Field	Number of publications
1	Journal of Religion, Spirituality and Aging	17
2	International Journal of Older People Nursing	11
3	Journal of Aging Studies	9
4	Ageing and Society	7
5	Aging and Mental Health	6
6	Educational Gerontology	5
7	The Gerontologist	4
8	International Journal of Aging and Human Development	4
9	Journal of Advanced Nursing	4
10	Adultspan Journal	3

The minimum number of publications among the ten most productive sources was three. The Journal of Religion, Spirituality, and Aging was the most productive, with 17 publications on gerotranscendence. The next two sources were the International Journal of Older People Nursing, with 11 publications, and the Journal of Aging Studies, with 9 publications.

#### The Most Cited Sources

**Fig. 7:**
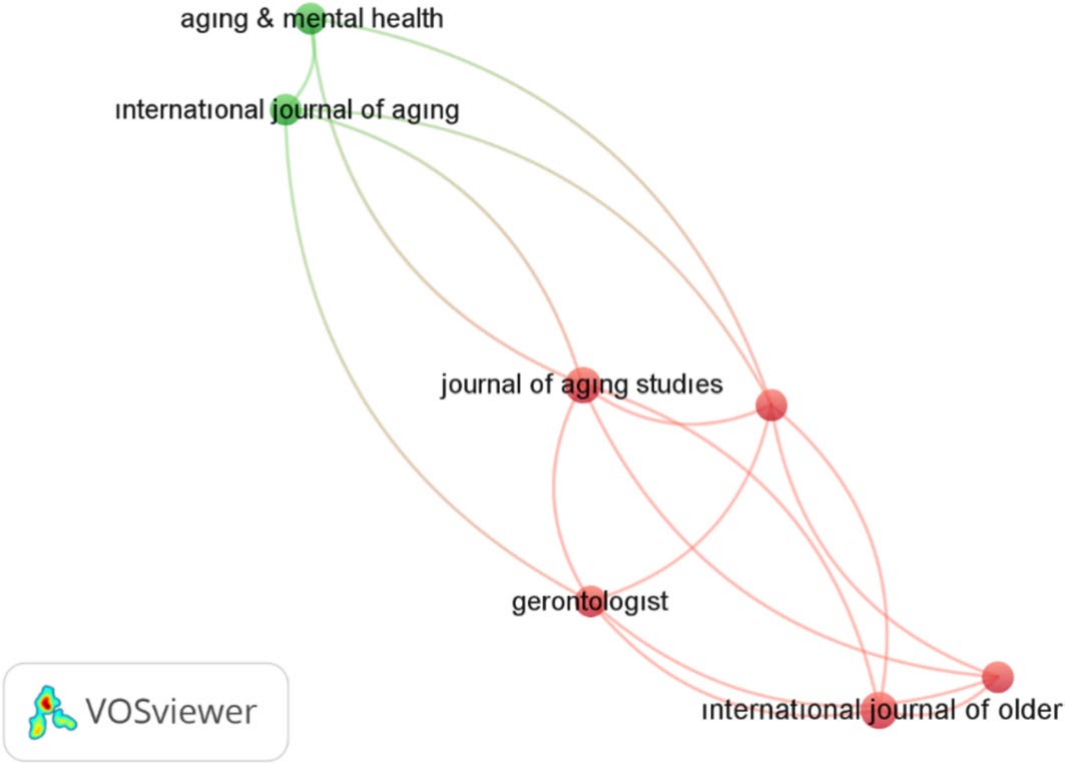
Distribution of the most cited journals on gerotranscendence (the name of some journals appears missing due to the application’s system)

Figure 7 shows the distribution of the most cited sources on gerotranscendence:

According to the density map, the Journal of Religion, Spirituality, and Aging is the most cited journal, with 17 citations across seven publications. Aging and Society and the Journal of Aging Studies rank as the second and third most cited sources, respectively. Educational Gerontology follows, with four citations across three publications.

### Themes of the Studies

**Fig. 8:**
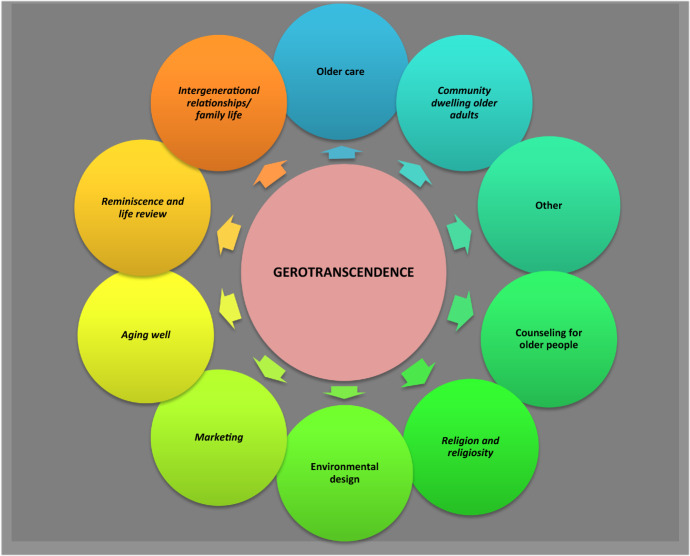
General themes of the articles derived from the contents

To understand the development of the studies as trends, the articles were categorized based on their content. Figure 8 presents a general overview of these themes.

#### Older Care

The findings indicate that Gerotranscendence Theory is applied in older care studies in four ways: a) the recognizing of signs by older care receivers (Wadensten, 2006; Wadensten, 2007b; Buchanan et al., 2015; Buchanan, 2016; Persson et al., 2018; Tip et al., 2024), b) recognizing signs by care professionals (Eriksson et al., 2024; Wadensten and Carlsson (2001, 2003, 2007a, 2007b), c) educational programs and alternative care perspectives (Wadensten & Carlsson, 2001, 2003, 2007a, 2007b; Ciriello & Karl, 2020; Jeong, Higgings & McMillan, 2011; Lin, Wang & Wang, 2015; Buchanan, 2016; Tip, Braam & van der Vaart, 2024d); and d) interventions for care recipients. The two main findings related to intervention activities are as follows. First, using a gerotranscendental perspective in activities has been found useful for older adults (Wadensten, 2005;2009; Chan, Moyle & Jones, 2019; Wang, 2011; Drummond & Carey, 2019).

Second, other activities, such as weekly thematic encounters (Abreu et al., 2023a, 2023b, 2023c, 2023d), music-making (Koivisto & Laes, 2022), art therapy (Stephenson, 2013), visual art programs (Rodrigues et al., 2018), and Tai Chi, may enhance the gerotranscendence level of individuals.

#### Community-Dwelling Older Adults

Some studies have identified signs of gerotranscendence in community-dwelling older adults (Wang et al., 2015; Ratan & de Vries, 2020; Bratun & Asaba, 2021). In addition, research has explored ethnic and cultural differences in the application of the theory (Jahnsen et al., 2021; George & Schlittler, 2024). Jönson and Magnusson (2001) reviewed empirical studies on Gerotranscendence Theory and noted that the theory is subject to “falsification criteria” since it has not yet been substantiated by empirical evidence. Similarly, Achenbaum (2006) questioned whether the theory holds universally across different cultures. Lewin (2001) studied gerotranscendence in Turkey and Sweden, focusing on religious and secular older adults from Iranian, Turkish, and Swedish backgrounds. The study supported a connection between gerotranscendence, religious thinking, and advanced age. Additional studies in American (George & Dixon, 2018), Indian (Bandyopadhyay & Singh, 2024), and African (Bohman, Van Wyk, & Ekman, 2011) contexts also emphasized the significance of cultural differences (Braam et al., 2006; Jeong et al., 2020; Wortman & Lewis, 2021).

Bratun et al. (2024) explored the relationship between gerotranscendence and retirement age. Their findings indicated that gerotranscendent older adults continue to find meaning in their work, even if they meet all criteria for retirement. This aligns with Tornstam’s (1992) assertion that not all aging processes and turning points are perceived as negative. Similarly, Dalheim-Englund et al. (2019) demonstrated that retirement does not necessarily result in disengagement from life.

The literature also includes studies that examine the relationship between gerotranscendence levels and age. George and Dixon (2018) found no significant relationship between age and gerotranscendence. However, other studies (Liu & Chen, 2022; Masui et al., 2024) suggest that being older and female is associated with higher levels of gerotranscendence. Kalavar et al. (2015) and Switsers et al. (2023) discovered that older adults who experience loneliness but feel less alone may exhibit signs of gerotranscendence. Washburn and Williams (2020) concluded that attitudes toward old age are linked to gerotranscendence.

#### Reminiscence and Life Review

Tornstam (1999) theoretically linked reminiscence with gerotranscendence, proposing its transformative function in the context of aging. Numerous studies have explored the role of reminiscence and life review in fostering gerotranscendence in older adults (Wadensten & Hägglund, 2006; Wadensten, 2010; Heinz et al., 2017; van Rhyn, Barwich & Donelly, 2022; Olafsson & Rykkje, 2022).

Nyman and Szymczynska (2016) also highlight the potential of reminiscence and life review activities, rooted in spiritual development and gerotranscendence, to enhance the well-being of individuals with dementia. Gallagher and Carey (2012) conducted reminiscence interviews with nursing students and older adults, identifying signs of gerotranscendence in the narratives. More recently, Mo (2024) incorporated reminiscence with metaverse technology, finding that this intervention significantly increased both the reminiscence function and the level of gerotranscendence among participants.

According to a scoping review by Kellehear and Garrido (2023), the concept of dying alongside is linked to personal changes associated with self/gerotranscendence. Read et al. (2014) explored the impact of adverse life events on gerotranscendence, finding that such events significantly influenced the level of cosmic transcendence. Other studies have further supported the role of life experiences and events as catalysts for gerotranscendence (Hui et al., 2014; Hoogland, 2015). Moody (2005) analyzed older adults’ narratives of dreams and identified signs of gerotranscendence within them. Retired narrative gerontologist William Randall (2023) also shared his personal aging journey, referring to this process as soulful aging in the context of gerotranscendence.

#### Narrative Analysis and Hermeneutics

Camcı and Çoban (2019) utilized the theory to interpret a Turkish poem, showcasing its relevance in literary analysis. Similarly, Zhang (2023) examined John Steinbeck’s The Red Pony through the lens of this theory. Gerotranscendence has also been employed as a theoretical framework for the analysis of religious texts, as demonstrated by Greenberger (2012) and Guyette (2018). Greenberger (2012) provides examples of gerotranscendence within biblical texts, while De Wet (2016) explored ancient religious sources in relation to the theory, further expanding its scope of application.

#### Intergenerational Relationships and Family Life

Bertram et al. (2018) applied this theory in a study with three generational samples, exploring the connection between the inner child and old age through intergenerational experiences, which also reflects self-transcendence. Similarly, Burr et al., (2015, 2019) investigated the play activities of older adults in the context of gerotranscendence. In Sabir’s (2015) study on attachment to family members, some participants’ responses were also linked to gerotranscendence.

Other studies have examined the role of forgiveness within family relationships, suggesting that both forgiveness and personality traits in marital contexts may be associated with wisdom as conceptualized in gerotranscendence (Brudek & Kaleta, 2023). Subsequently, further research demonstrated that forgiveness serves as a mediating factor between gerotranscendence and wisdom (Brudek et al., 2024).

#### Counseling for Older Adults

Research has indicated that counseling interventions for older adults offer a renewed perspective on service delivery tailored to this demographic (Degges-White, 2005; George & Dixon, 2018). Furthermore, the Gerotranscendence Theory has been applied to counseling within romantic relationships, highlighting how individuals’ views on sexuality and relational dynamics may evolve with age. This shift can be conceptualized through the lens of psychosexual gerotranscendence (Fleckenstein & Cox II, 2014).

Weiss (2014) draws parallels between gerotranscendence and post-traumatic growth, emphasizing their shared emphasis on psychological transformation following significant life events. Similarly, Lee, Choi, and Lee (2020) suggest that in advanced old age, individuals confront the reality of death while continuing to experience personal development, as conceptualized within the framework of gerotranscendence.

#### Religion and Religiosity

Although Tornstam did not explicitly incorporate religion into his formulation of gerotranscendence, numerous studies have explored its intersection with religiosity in the aging process (Dalby, 2006). Jewell (2014a, 2014b) examined the theory’s universality and its relationship to age, highlighting that only a limited number of studies have substantiated the validity of cosmic transcendence. She further critiqued the theory for its omission of the concept of God, suggesting a potential limitation in its applicability to spiritually oriented populations. In line with this perspective, Abreu et al. (2023d) emphasized the need for further empirical research on the religious dimensions of gerotranscendence.

A study by Braam et al. (2006) showed that the cosmic transcendence were significantly higher among nonreligious individuals aged 75 and older, particularly among women, compared to their younger, religious, and male counterparts.

In contrast, a study conducted in Portugal by Abreu, Ribeiro, and Araujo (2023a) revealed that gerotranscendence levels were higher among religious participants than their nonreligious peers. Patrick et al. (2021) utilized Gerotranscendence Theory to explain age-related changes in religious doubt. Additional studies examining the relationship between gerotranscendence and religiosity have focused on terminal cancer patients (Balducci, 2019), individuals across varying age groups and transitional life stages (Silverstein & Bengston, 2018), Methodists (Jewell, 2010), and evangelical older adults (Yount, 2008).

Hong (2021) highlighted conceptual parallels between Gerotranscendence Theory and Confucianism, proposing that Confucian philosophy may offer an alternative lens through which to understand spirituality in later life. Braam et al. (2016) examined the association between religious beliefs and cosmic transcendence, while Brudek (2024) found that religious meaning systems foster both wisdom and gerotranscendence. Upenieks (2023) further explored gerotranscendence through both spiritual and religious frameworks. Le (2008) emphasized that mystical experiences, coupled with advanced age, are more strongly associated with transcendental wisdom. Additionally, Abreu et al. (2023b) compared the constructs of gerotranscendence and self-transcendence, contributing to the ongoing discourse on the conceptual boundaries and intersections between these terms.

#### Perception of Aging Well

Since Tornstam (2005, 2011) posited a connection between gerotranscendence and life satisfaction, a growing body of research has examined its relationship with various aspects of aging. These include successful aging (Troutman-Jordan & Staples, 2014; Gutiérrez et al., 2018a, 2018b; Gutiérrez, Tomás, & Calatayud, 2018; Khan & Shah, 2023), active aging (Adams, 2004; Topaz et al., 2014; Lim & Thompson, 2016; Wong, Low & Yap, 2016; Dehkordi et al., 2021), mindful aging (Nilsson et al., 1996), and healthy aging (Cho & Cheon, 2023; Holmgren & Ahlström, 2023; Kim & Seo, 2022). Furthermore, the theory has been linked to psychological well-being (Brudek et al., 2023; Gondo et al., 2012; Wang et al., 2011) and oral health-related quality of life (Koudai et al., 2005; Das, Yavagal & Nandeeschumar, 2023). Palmér et al. (2019) also found that the self-perception of aging among 18 healthy older adults was positively associated with gerotranscendence.

#### Environmental Design

Afacan (2021, 2024) examined the influence of biophilic design on gerotranscendence in the Turkey context, revealing that exposure to nature significantly enhances the cosmic dimension of gerotranscendence. Similarly, Wang and Hsu (2024) explored the application of Gerotranscendence Theory into the development of interactive art systems for older adults, underscoring its growing relevance in the domain of gerotechnology. This study demonstrates that this theory has extended its influence in the field of gerotechnology. Since Gerotranscendence Theory proposes body transcendence, other authors have suggested that it may also explain aging in place and its relation to aging self-concept among older adults with disabilities (Wiles & Allen, 2010).

#### Marketing Context

A recent study conducted in France by Bourcier-Béquaert et al. (2024) demonstrated that incorporating the Gerotranscendence Theory into advertising strategies enhances the appeal of advertisements by promoting a positive redefinition of old age.

#### Other Quantitative Studies

The development and cross-cultural adaptation of gerotranscendence measurement scales have been conducted in several countries, including translations into Polish (Brudek, 2021), Persian (Asiri et al., 2019), and Japanese (Hoshino et al., 2012). Furthermore, new assessment tools grounded in Gerotranscendence Theory have been proposed. For instance, Nilsson et al. (2015) introduced the concept of “mindful sustainable ageing” as a novel framework. Dewitte and Desutzer (2021) explored reflectivity in adults aged over 75, while Brudek and Steuden (2017) investigated the factors influencing self-dignity in the context of gerotranscendence. Additionally, Timoszyk-Tomczak and Bujaska (2019) developed the Transcendent and Transcendental Time Perspective Inventory, further enriching the psychometric tools available for research in this domain.

## Discussion

The analysis reveals that the Theory of Gerotranscendence has been applied across a wide range of subfields within gerontology and aging-related research. Numerous review studies have underscored the theory’s relevance and practical utility, both in academic inquiry and in care-related contexts. For instance, Rajani and Jawaid (2015) emphasized that gerotranscendence is intimately connected to the subjective experience of aging, particularly within caregiving practices for older adults. Their findings suggest that the theory offers a valuable framework for understanding how aging individuals perceive and adapt to the aging process.

Since Tornstam (1996) proposed gerotranscendence as an alternative framework for evaluating older care recipients, numerous studies have explored its applicability. The findings of these studies support the idea that older care is multifaceted—not only focused on the needs of care recipients, but also influenced by care providers’ perceptions of aging and their relationships with older people. Additionally, interventional studies suggest that Tornstam’s theory can be effectively integrated into caregiving practices, both in institutional settings and community-based care (Tornstam, 2005, 2011).

Tornstam (2005) critiqued the dominant “misery perspective” in aging research, which tends to focus disproportionately on decline, dependency, and loss, and highlighted the limitations of an overreliance on quantitative methodologies that may inadequately reflect the complexity of aging as a lived experience. In response, he proposed the Theory of Gerotranscendence as a more comprehensive and humanistic framework for understanding aging, one that emphasizes personal growth, spiritual development, and a redefinition of self and relationships in later life. Although his empirical work primarily explored cosmic transcendence using quantitative methods, Tornstam (2005, 2011) and Hyse and Tornstam (2009) acknowledged the inherent limitations of such approaches and stressed that gerotranscendence is not a uniform process—individuals may display varying degrees and dimensions of transcendence. These assertions have been substantiated by subsequent studies, which confirm the variability and subjectivity in how older adults experience gerotranscendence.

One primary criticism of the Theory of Gerotranscendence is its potential cultural specificity, with some questioning its generalizability across diverse settings and populations (Rajani & Jawaid, 2015). However, Tornstam (2005, 2011) argued that the theory’s core concepts remain relevant across different cultural contexts, as long as they are adapted to the specific needs and values of each society. This review found evidence supporting the theory’s applicability in various cultural contexts, suggesting that its fundamental principles can transcend cultural boundaries when appropriately tailored to local customs and experiences of aging.

Another important theme in the Gerotranscendence Theory is the influence of life crises and turning points. Although some scholars have criticized Tornstam for restricting the theory’s focus to old age (Rajani & Jawaid, 2015), Tornstam acknowledged that gerotranscendence could occur at any stage of life, particularly in response to significant crises (Tornstam, 2011). Our findings support this perspective, with several studies pointing to instances in which gerotranscendence occurs outside the context of old age.

The intersection between gerotranscendence and spirituality has been explored in various studies. Atchley (2009) offers a broad definition of spirituality that encompasses both religiosity and a more general search for existential meaning. Tornstam’s theory aligns with this inclusive perspective, proposing that gerotranscendence involves both religious and nonreligious experiences. This broadens the scope of the theory and makes it relevant to a diverse range of individuals regardless of their religious beliefs.

The Theory of Gerotranscendence emerged in the late 1980s and early 1990s, coinciding with a shift in aging research toward lifespan developmental perspectives (Abreu et al., 2023b). A related framework is the Self-Transcendence Theory (Reed, 1991), which shares similar developmental roots, drawing on the work of theorists such as Peck (1956), Erikson (1982), and Chinen (1986). The primary distinction between the two theories lies in their disciplinary origins: Self-Transcendence Theory was developed within the field of nursing science, whereas Gerotranscendence Theory originated in gerontology and was influenced by the Disengagement Theory of Aging (Cumming & Henry, 1961). However, findings from the present study suggest that Tornstam’s conceptualization has increasingly been applied in health sciences and nursing. Further research that examines both theoretical foundations and evolving scopes of application could provide greater clarity regarding the distinctions and overlaps between these two perspectives.

## Limitations

This study aimed to evaluate and visualize publication trends related to Lars Tornstam’s Gerotranscendence Theory. However, several limitations should be acknowledged. First, the literature search was confined to the Web of Science database, and findings were not cross-referenced with other databases such as Scopus or Google Scholar, which may have offered a more comprehensive overview of both published and current literature. Second, the analysis was limited to articles published in English, potentially excluding relevant studies in other languages.

## Conclusion

In conclusion, the application of the Gerotranscendence Theory continues to expand in gerontology, and aging research, demonstrating its value as a framework for understanding the complex and multidimensional aspects of aging. Although the theory has faced criticism—particularly regarding its cultural specificity and age-related focus—findings from this study support Tornstam’s assertion that gerotranscendence is a flexible and adaptable concept, applicable across diverse populations and settings. As the field evolves, future research should further investigate the intersection of gerotranscendence with other aging theories, assess its cultural relevance, and explore its potential to enhance well-being among older adults in both institutional and community contexts.

The individualized and crisis-responsive nature of gerotranscendence, along with its potential for cross-cultural applicability, has contributed to its increasing use in older adult care and positive aging interventions. This personalized perspective recognizes that gerotranscendence may manifest uniquely based on an individual’s life experiences, crises, and cultural background. Notably, the theory accommodates a broad spectrum of spiritual and non-spiritual orientations, providing a unifying framework for individuals across diverse belief systems.

This flexibility is invaluable for practitioners. Understanding that Gerotranscendence may emerge to varying degrees depending on the individual can guide care planning and intervention. By assessing the extent to which gerotranscendence is present in their clients, practitioners can tailor their approaches to better support older adults in achieving a sense of meaning, purpose, and transcendence during later stages of life. This individualized, person-centered care model can enhance the well-being of aging individuals, whether in institutional settings or within the broader community.

## Data Availability

The original data and results generated in this study are available from the corresponding author upon reasonable request.
